# Carbon footprint of non-melanoma skin cancer surgery

**DOI:** 10.1093/bjsopen/zrae084

**Published:** 2024-10-17

**Authors:** Ky-Leigh Ang, Matthew Jovic, Ian Malin, Stephen R Ali, Sairan Whitaker, Iain S Whitaker

**Affiliations:** Oxford University Clinical Academic Graduate School, University of Oxford, Oxford, UK; Welsh Centre for Burns and Plastic Surgery, Morriston Hospital, Swansea, UK; Reconstructive Surgery and Regenerative Medicine Research Centre (ReconRegen), Institute of Life Sciences, Swansea University Medical School, Swansea, UK; Welsh Centre for Burns and Plastic Surgery, Morriston Hospital, Swansea, UK; Oxford University Clinical Academic Graduate School, University of Oxford, Oxford, UK; Welsh Centre for Burns and Plastic Surgery, Morriston Hospital, Swansea, UK; Reconstructive Surgery and Regenerative Medicine Research Centre (ReconRegen), Institute of Life Sciences, Swansea University Medical School, Swansea, UK; Dermatology Department, Singleton Hospital, Swansea, UK; Oxford University Clinical Academic Graduate School, University of Oxford, Oxford, UK; Welsh Centre for Burns and Plastic Surgery, Morriston Hospital, Swansea, UK; Reconstructive Surgery and Regenerative Medicine Research Centre (ReconRegen), Institute of Life Sciences, Swansea University Medical School, Swansea, UK

## Abstract

**Background:**

Climate change poses a significant global health threat and healthcare, including surgery, contributes to greenhouse gas emissions. Efforts have been made to promote sustainability in surgery, but the literature on sustainability in plastic surgery remains limited.

**Methods:**

A life-cycle analysis was used to assess and quantify the environmental emissions associated with three distinct reconstructive methods utilized in non-melanoma skin cancer surgery: direct closure, split-thickness skin graft, and full-thickness skin graft. Analyses were conducted in March 2023 in Morriston Hospital, Swansea, UK. The carbon footprints for non-melanoma skin cancer surgery in England and Wales were then estimated.

**Results:**

The mean carbon emissions for non-melanoma skin cancer surgery ranged from 29.82 to 34.31 kgCO₂eq. Theatre energy consumption (4.29–8.76 kgCO₂eq) and consumables (16.87 kgCO₂eq) were significant contributors. Waste produced during non-melanoma skin cancer surgery accounted for 1.31 kgCO₂eq and sterilization of reusable surgical instruments resulted in 1.92 kgCO₂eq of carbon emissions. Meanwhile, transportation, dressings, pharmaceuticals, and laundry accounted for 0.57, 2.65, 1.85, and 0.38 kgCO₂eq respectively. The excision of non-melanoma skin cancer with direct closure (19.29–22.41 kgCO₂eq) resulted in the lowest carbon emissions compared with excision with split-thickness skin graft (43.80–49.06 kgCO₂eq) and full-thickness skin graft (31.58–37.02 kgCO₂eq). In 2021, it was estimated that non-melanoma skin cancer surgery had an annual carbon footprint of 306 775 kgCO₂eq in Wales and 4 402 650 kgCO₂eq in England. It was possible to predict that, by 2035, carbon emissions from non-melanoma skin cancer surgery will account for 388 927 kgCO₂eq in Wales and 5 419 770 kgCO₂eq in England.

**Conclusion:**

This study highlights the environmental impact of non-melanoma skin cancer in plastic surgery departments and emphasizes the need for sustainable practices. Collaboration between surgeons and policymakers is essential and further data collection is recommended for better analysis.

## Teaser

The mean carbon emissions for NMSC range from 29.82 to 34.31 kgCO₂eq, with consumables being the most significant contributing factor. A split-thickness skin graft results in larger carbon emissions compared with direct closure and a full-thickness skin graft. By 2035, it is estimated that NMSC surgery will account for 388 927 kgCO₂eq in Wales and 5 419 770 kgCO₂eq in England.

## Introduction

The Lancet Climate Change Commission has recognized climate change as one of the biggest global health threats of the 21st century^1^. Climate change threatens the foundation of good health, with direct and immediate consequences for patients, the public, and the National Health Service (NHS)^2–4^. The impact of climate change on public health is increasingly apparent through extreme weather events, food and water insecurity, changes in disease vector ecology, and forced migration^5–8^. Such consequences may include an increase in the rate of surgical-site infections^9^, cancellation of elective procedures^10,11^, and an increased incidence of malignant melanoma^12^.

The United Nations^13^ has reported that urgent climate action must be undertaken now to secure a habitable and liveable future.

The role of healthcare in exacerbating climate change is well documented, contributing to 4–6% of global carbon emissions^8^. If the healthcare sector was a country, it would rank fifth among global polluters. In the UK, the NHS is the largest public sector greenhouse gas (GHG) emitter, responsible for 25% of public sector carbon dioxide (CO_2_) emissions and approximately 5% of all carbon emissions in the UK^14,15^.

The NHS is becoming increasingly aware about the implications of climate change for health and social care. In October 2020, the NHS became the world’s first health service to set a target for net zero carbon emissions by 2045, through its Greener NHS campaign^14^. Surgery is the largest contributor to NHS GHG emissions, with operating theatres being up to six times more energy intensive than other hospital areas^16^ and generating 50–70% of total hospital waste^17,18^.

To achieve a net zero carbon NHS, surgical teams must be engaged in sustainable practices. Initiatives like the ‘Greener Operations: Sustainable Peri-Operative Practice’ Priority Setting Partnership (PSP), established in early 2021 with inputs from patients, carers, and healthcare professionals^19^, have identified the priority areas where further research is needed. Additionally, the Royal College of Surgeons of England and the Royal College of Surgeons of Edinburgh have also produced guidelines for sustainable practices in the operating theatre^20,21^. However, operations are only a fraction of surgery and other aspects of surgery, such as patient pathways, anaesthesia, preoperative care, and postoperative care, should also be considered.

Carbon footprinting is a quantitative method for measuring environmental impact by accounting for direct and indirect GHG emissions associated with a particular product, process, or service. This includes six GHGs covered by the Kyoto Protocol: CO_2_, methane, nitrous oxide, hydrofluorocarbons, perfluorocarbons, and sulphurhexafluoride^22^. Emission data for all six GHGs covered can be reported together as CO_2_ equivalent (CO_2_eq).

While various carbon footprinting studies with varying methodologies have emerged in the medical literature^23^, studies focused on quantifying the sustainability of plastic surgery procedures remain under-represented^24^. Life-cycle assessments of individual procedures are required to identify the most effective environmental intervention to maximize efficiency^23^. Non-melanoma skin cancer (NMSC), such as basal cell carcinoma and cutaneous squamous cell carcinoma, is the UK’s most prevalent cancer, with global rates on the rise^25^. Surgical excision is the primary treatment for NMSC^26,27^, making it an ideal target for emission reduction efforts. In the UK, plastic surgeons handle the majority of complex and high-risk NMSC cases. Hence, the aim of this study was to determine the magnitude of GHG emissions attributable to NMSC surgery in a plastic surgery centre.

## Methods

**Fig. 1:**
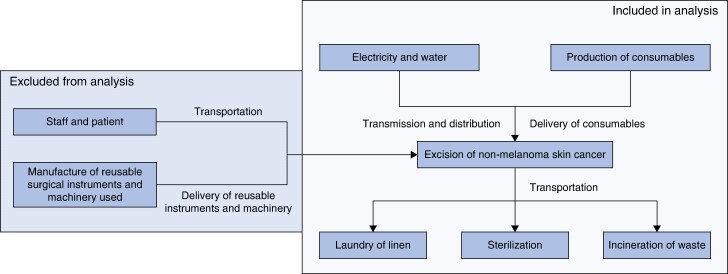
Inclusion and exclusion criteria for analysis

This study took place in the Welsh Centre for Burns and Plastic Surgery (WCBPS) in Morriston Hospital, Swansea, UK, aiming to evaluate the carbon footprint and identify major carbon hotspots when a patient attended the hospital for day surgery. Only the activities carried out during the surgery were considered (*Fig. 1*). The WCBPS’s skin cancer pathway is illustrated in *Fig. S1*.

Carbon footprints were estimated in accordance with the Greenhouse Gas Accounting Sector Guidance for Pharmaceutical Products and Medical Devices^28^. A hybrid approach was adopted, combining both a processed-based (bottom-up) carbon footprinting approach and an environmentally extended input-output (top-down) analysis. Emission factors were sourced from the Department for Business, Energy & Industrial Strategy (BEIS) Greenhouse Gas Conversion Factors for Company Reporting^29^, the Inventory of Carbon and Energy (ICE) Database Version 3^30^, studies of healthcare waste^31^, sterilization of reusable surgical instruments^32^, personal protective equipment^33^, and laundry^34^, the WorldStainless Carbon Factors data set^35^, and the Greener NHS emission factors 2020–2021^36^ (*Table S1*).

The resulting carbon footprints are expressed as CO_2_eq and summarize the direct GHG emissions (for example anaesthetic gases) and indirect emissions (for example electricity, water, and consumables (for example disposable gowns, drapes, and gallipots)). In the case of day surgery, no direct emissions occurred, as there was no use of anaesthetic gases.

A material flow analysis (MFA) was conducted to track all consumables used during procedures over a 3-week interval in March 2023. MFA is a widely recognized approach used to evaluate the movement and quantities of materials throughout their life cycles, while measuring environmental emissions^37,38^. This method has been extensively used in previous studies assessing the life cycle of a procedure^39–46^. Data collection was prospective to minimize recall and information biases and the 3-week interval was deemed representative of NMSC patients treated at the WCBPS.

The study took place in a designated operating theatre in the Plastic Surgery Treatment Centre (PSTC) at the WCBPS. The PSTC is a facility that is dedicated to the provision of day-care skin cancer surgery. The results were used to calculate the mean carbon footprint of NMSC excision. A subgroup analysis of three different wound closure methods of NMSC surgery was performed: direct closure (DC), split-thickness skin graft (STSG), and full-thickness skin graft (FTSG). No NMSC excisions closed using local flaps were observed during the study interval.

The mean emissions were extrapolated to project carbon emission trends related to NMSC surgery. Several assumptions were made for forecasting. First, the distribution of patients treated by specialty was assumed to follow the same pattern as observed in a previous study^47^. The percentage of reconstructive procedures for dermatology was adopted from another relevant study^48^. Hospital episode data^49^ were used to estimate the proportion of cases treated by each reconstructive method and the number of NMSC procedures carried out by different specialties. To predict the number of NMSC cases in England, the Get Data Out: Skin Tumours data set^50^ was used and, to predict the number of NMSC cases in Wales, the Welsh Cancer Intelligence and Surveillance Unit database^51^ was used. To determine the proportion of closure methods used in NMSC surgery, data from a prior study were utilized^48^. Additionally, as the carbon footprint for local flaps was not directly calculated, the emissions using a model based on the results obtained from the skin graft emissions were estimated.

### Boundary setting

Emissions from primary sectors, such as building energy use and procurement, encompassing both direct emissions from building energy use and indirect procurement emissions, were included (for example emissions associated with production, consumption, and disposal of all goods and services consumed during the surgery). Emissions related to sterilization of reusable instruments and laundry were also included (*Fig. 1*).

In accordance with the Publicly Available Specification (PAS2050), certain GHG emissions were excluded, including building and construction, costs of capital equipment (for example loupes, operating tables, and reusable surgical instruments), food and beverages for staff, scientific research, staff training, and immaterial emission sources (those anticipated to be less than 1% of the total emissions). Patient and staff travel emissions were also excluded.

Water use was based on an observational study of 35 scrubbing episodes in the operating theatre. This approach allowed for a comprehensive calculation of the actual amount of water used during scrubbing episodes. A post-hoc analysis found that the carbon emissions from water used during scrubbing accounted for less than 1% of total emissions and hence this was excluded from the main analysis (*Appendix S1*).

### Approach and assumptions

Several informed assumptions within any carbon footprinting methodology were used as outlined below.

For transportation, it was assumed that all items were shipped to the UK from their country of origin via sea, as sea freight accounts for more than 90% of all goods^52^. Two hundred kilometres of travel by road via heavy good vehicles in the country of origin and in the UK was included, with an additional 20 km at either end of each journey via courier. All transportation distances were estimated using the online Pier2Pier tool^53^ (*Table S2*). A similar method was used in a previous study^33^.

Sterilization of reusable instruments and laundry of linen used by patients is carried out off-site. Distances were determined using Google Maps (http://maps.google.co.uk). The distances made for one return journey via a courier were calculated.

Energy was estimated based on hospital data from 2022 and is based upon the proportion of floor space required by the operating theatre used and the proportion of time for which these areas are in use for this purpose. As there was no meter reading specific to the operating theatre, the energy use was estimated to be three to six times that of the mean energy use for a given floor space within the hospital^16^. A similar approach was taken in previous studies^39^.

The weight of waste from NMSC surgery was measured and apportioned to the domestic, clinical waste, sharps, or recycle streams. The carbon emissions from waste and sterilization were estimated from the studies of Rizan *et al*.^31,32^ on clinical waste and sterilization.

## Results

Over the 3-week data collection interval, 13 patients had NMSC excision with DC, 7 patients had NMSC excision with STSG, and 12 patients had NMSC excision with FTSG. The contribution of each sector is summarized in *Table 1*.

**Table 1:** Greenhouse gas emissions attributable to an individual patient undergoing non-melanoma skin cancer excision

Sector	Carbon footprint (95% c.i.) (kgCO_2_eq)	Percentage of total greenhouse gas emissions
**Total**		
Building energy (theatre)	4.27 (3.72,4.82)*/8.76 (7.59,9.94)†	14.32*/25.53†
Consumables	16.87 (14.78,18.96)	56.57*/49.17†
Dressings	2.65 (1.75,3.54)	8.89*/7.72†
Waste products	1.31 (1.22,1.39)	4.39*/3.82†
Sterilization	1.92 (1.87,1.97)	6.44*/5.60†
Pharmaceuticals	1.85 (1.43,2.28)	6.20*/5.39†
Laundry	0.38 (0.37,0.40)	1.27*/1.11†
Transportation	0.57 (0.53,0.62)	1.91*/1.66†
Total carbon footprint	29.82 (26.40,33.24)*/34.31 (30.46,38.17)†	100
**Direct closure**		
Building energy (theatre)	3.12 (2.55,3.69)*/6.24 (5.10,7.38)†	16.17*/27.83†
Consumables	10.63 (9.64,11.61)	55.01*/47.41†
Dressings	0.58 (0.34,0.83)	3.01*/2.59†
Waste products	1.12 (1.02,1.22)	5.80*/5.00†
Sterilization	1.78 (1.78,1.78)	9.22*/7.94†
Pharmaceuticals	1.23 (0.87,1.58)	6.37*/5.49†
Laundry	0.36 (0.34,0.38)	1.87*/1.61†
Transportation	0.48 (0.43,0.53)	2.49*/2.14†
Total carbon footprint	19.29 (17.82,20.76)*/22.41 (20.52,24.30)†	100
**Full-thickness skin graft**		
Building energy (theatre)	4.83 (4.14,5.53)*/10.28 (8.66,11.90)†	15.30*/27.77†
Consumables	17.96 (16.82,19.09)	56.89*/48.51†
Dressings	2.44 (1.74,3.14)	7.73*/6.59†
Waste products	1.27 (1.14,1.40)	4.02*/3.43†
Sterilization	1.78 (1.78,1.78)	5.64*/4.81†
Pharmaceuticals	2.30 (1.37,3.23)	7.29*/6.21†
Laundry	0.39 (0.37,0.41)	1.24*/1.05†
Transportation	0.60 (0.56,0.64)	1.90*/1.62†
Total carbon footprint	31.58 (30.37,32.78)*/37.02 (35.17,38.87)†	100
**Split-thickness skin graft**		
Building energy (theatre)	5.25 (4.03,6.48)*/10.51 (8.06,12.96)†	11.99*/21.43†
Consumables	25.18 (23.51,26.86)	57.41*/51.35†
Dressings	6.69 (5.22,8.15)	15.28*/13.64†
Waste products	1.55 (1.43,1.68)	3.54*/3.16†
Sterilization	2.14 (2.14,2.14)	4.89*/4.36†
Pharmaceuticals	1.92 (1.20,2.65)	4.39*/3.92†
Laundry	0.36 (0.34,0.38)	0.82*/0.73†
Transportation	0.69 (0.66,0.72)	1.57*/1.41†
Total carbon footprint	43.80 (42.68,44.91)*/49.06 (46.80,51.30)†	100

*Modelled under the assumption that the building energy use was three times that of the mean energy for a given floor space within the hospital. †Modelled under the assumption that the building energy use was six times that of the mean energy for a given floor space within the hospital.

The mean carbon emissions for NMSC excision in the WCBPS ranged from 29.82 (95% c.i. 26.40 to 33.24) to 34.31 (95% c.i. 30.46 to 38.17) kgCO₂eq, with theatre energy use accounting for 4.27 (95% c.i. 3.72 to 4.82) to 8.76 (95% c.i. 7.59 to 9.94) kgCO₂eq and consumables contributing 16.87 (95% c.i. 14.78 to 18.96) kgCO₂eq. Waste produced during NMSC surgery accounted for 1.31 (95% c.i. 1.22 to 1.39) kgCO₂eq and sterilization of reusable surgical instruments resulted in 1.92 (95% c.i. 1.87 to 1.97) kgCO₂eq of carbon emissions. Transportation, dressings, pharmaceuticals, and laundry accounted for 0.57 (95% c.i. 0.53 to 0.62), 2.65 (95% c.i. 1.75 to 3.35), 1.85 (95% c.i. 1.43 to 2.28), and 0.38 (95% c.i. 0.37 to 0.40) kgCO₂eq respectively.

**Fig. 2:**
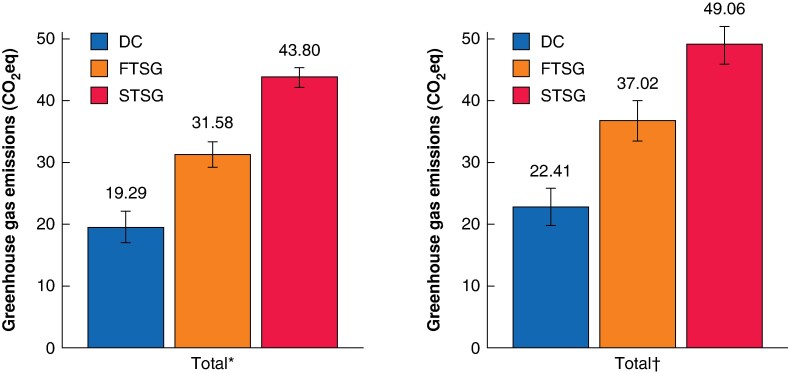
Total greenhouse gas emissions of non-melanoma skin cancer excision by closure type *Modelled under the assumption that the building energy use was three times that of the mean energy for a given floor space within the hospital. †Modelled under the assumption that the building energy use was six times that of the mean energy for a given floor space within the hospital. DC, direct closure; FTSG, full-thickness skin graft; STSG, split-thickness skin graft.

DC had the lowest emissions (19.29 (95% c.i. 17.82 to 20.76) to 22.41 (95% c.i. 20.52 to 24.30) kgCO₂eq), FTSG had higher emissions (31.58 (95% c.i. 30.37 to 32.78) to 37.02 (95% c.i. 35.17 to 38.87) kgCO₂eq), and STSG had the highest emissions (43.80 (95% c.i. 42.68 to 44.91) to 49.06 (95% c.i. 46.80 to 51.30) kgCO₂eq) (*Fig. 2*).

**Fig. 3:**
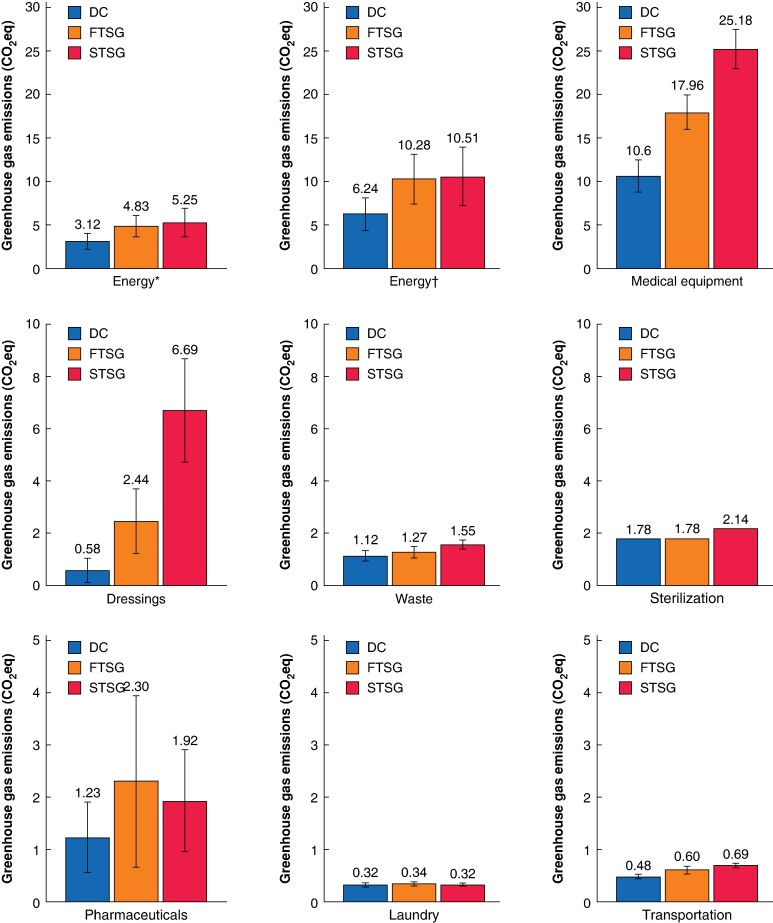
Greenhouse gas emissions of various sectors of non-melanoma skin cancer excision by closure type *Modelled under the assumption that the building energy use was three times that of the mean energy for a given floor space within the hospital. †Modelled under the assumption that the building energy use was six times that of the mean energy for a given floor space within the hospital. DC, direct closure; FTSG, full-thickness skin graft; STSG, split-thickness skin graft.

Theatre energy use accounted for a significant portion of carbon emissions from all NMSC excision procedures, ranging from 3.12 (95% c.i. 2.55 to 3.69) kgCO₂eq in NMSC surgery with DC (modelled under the assumption that building energy use was three times that of mean energy for a given floor space) to 10.51 (95% c.i. 8.06 to 12.96) kgCO₂eq in NMSC surgery with STSG (modelled under the assumption that building energy use was six times that of mean energy for a given floor space). See *Fig. 3*. Consumables contributed the highest proportion of carbon emissions across all wound closure methods: 10.63 (95% c.i. 9.64 to 11.61) kgCO₂eq for DC, 17.96 (95% c.i. 16.82 to 19.09) kgCO₂eq for FTSG, and 25.18 (95% c.i. 23.51 to 26.86) kgCO₂eq for STSG (*Table 1*).

Extrapolating the emission data obtained in this study to the 12 142 NMSC surgeries in Wales and the 170 490 NMSC surgeries in England that were carried out in 2021 gave an estimated annual carbon footprint of NMSC surgery in Wales and England of 306 775 and 4 402 650 kgCO₂eq respectively (*Fig. S2*). The details of the forecasting of the carbon footprint associated with NMSC excision surgery are included in *Table S3*. Based on the forecasts of NMSC incidence, by the year 2035, it is estimated that carbon emissions from NMSC surgery will account for 388 927 kgCO₂eq in Wales and 5 419 770 kgCO₂eq in England. Of these emissions, in Wales, it is estimated that 47 632 kgCO₂eq will be from FTSG closure, 33 467 kgCO₂eq will be from STSG closure, 62 608 kgCO₂eq will be from flap closure, and 207 495 kgCO₂eq will be from DC (*Fig. S3*). In comparison, the projected figures for England suggest 664 956 kgCO₂eq will be from FTSG closure, 467 206 kgCO₂eq will be from STSG closure, 864 244 kgCO₂eq will be from flap closure, and 3 423 365 kgCO₂eq will be from DC (*Fig. S4*).

## Discussion

Climate change is a major global health crisis^1^, with the healthcare sector that includes the NHS contributing significantly to GHG emissions^2–4^. NMSC is the most common cancer in the UK, with incidence increasing worldwide^25,54,55^. Therefore, addressing the environmental impact of NMSC surgery is essential to identify areas for improvement.

This study quantified the carbon footprint associated with day cases of NMSC excisions and three wound closure methods in a plastic surgery centre. The mean carbon footprint of NMSC excision ranged from 29.76 to 34.26 kgCO₂eq. This is equivalent to a 4.77 to 6.10 km drive in a medium-sized petrol car. Consumables and building energy use were the primary contributors to the carbon footprint, with DC exhibiting the lowest mean carbon emissions compared with STSG and FTSG. Extrapolating the findings to national NMSC incidence forecasts suggests a potential 25% increase in carbon emissions from NMSC surgery in England and Wales by 2035.

The NHS has committed to achieving net zero carbon emissions by 2045^14^ and the Greener NHS programme, initiated in 2020, aims to address climate change, while enhancing public health and reducing costs^56^. However, the lack of life-cycle analyses within the NHS has hindered progress towards achieving a net zero NHS^39,46,57–61^. Implementing more sustainable models of care is crucial^14^ and this requires the urgent identification of feasible pathways and strategies.

Carbon emissions from NMSC surgery are projected to increase significantly in the coming decade if current practices persist, underscoring the urgency of immediate action to align NMSC surgery with the NHS’s net zero carbon ambition. This is the first study, to the best of the authors’ knowledge, to quantify the carbon footprint of NMSC surgery in a plastic surgery department. There are limited data in the literature on the carbon footprint of plastic surgery^24^ or dermatological oncology^62^. Previous studies have shown that the carbon footprint of various surgical procedures^24,39,42,63–65^ ranges from 6 to 814 kgCO₂eq. Direct comparisons between procedures are challenging due to differences in study boundaries^63^.

The procurement of surgical supplies emerged as a major source of emissions from NMSC surgery, with disposable consumables (49–57%), dressings (8–9%), and pharmaceuticals (5–6%) contributing significantly. This finding aligns with other surgical carbon footprinting studies^39,63^, emphasizing the need for alternatives, such as reusable surgical equipment, to reduce GHG emissions.

Single-use items are prominent contributors to GHG emissions from operating theatres^63^ and generate landfill waste, releasing methane gas. Reusable surgical equipment is a low-carbon alternative and can reduce the carbon footprint by 50–97%^63,66–70^. For instance, reusable surgical gowns have 66% lower GHG emissions than disposable gowns^70^. Additionally, implementation of reusable surgical instruments is highly feasible^71^. Multidisciplinary surgical teams can further minimize consumable use by opening items only when needed and a hybrid approach of combining reusable and single-use items may be considered when appropriate.

Theatre energy use is a significant contributor to overall carbon emissions, closely tied to the duration of operative procedures^24^. NMSC surgery with DC exhibited the lowest theatre energy use, whereas more complex and lengthy closures using STSG were associated with higher energy consumption. Sterilization of instruments accounted for approximately 6% of carbon emissions from NMSC surgery, suggesting potential reductions through condensing instrument sets by removing rarely used instruments.

Promoting carbon literacy among healthcare staff is essential to achieve sustainability goals^72^. A deep understanding of carbon footprints, GHG emissions, and their environmental impact empowers staff to make informed decisions and take actions to reduce their environmental footprints, while enhancing engagement and morale. Collaboration between the surgical community and industry is essential to develop viable solutions that maximize surgical efficiency, while minimizing environmental impact^73^.

Anaesthetic gases contribute approximately 525 ktCO₂eq to NHS emissions, with 200 ktCO₂eq attributed to surgical use alone^74^. Notably, this study only includes patients who attended the day-surgery unit and did not require any general anaesthetic, highlighting the substantial environmental impact of NMSC surgery, even in the absence of typical volatile anaesthetic gases.

This study estimates the national carbon emissions associated with NMSC surgery, providing insights into carbon hotspots of NMSC surgery. By quantifying the carbon footprint of NMSC surgery, a crucial knowledge gap has been addressed, laying a foundation to enhance the sustainability of NMSC surgery within an operating theatre.

This study also serves as a benchmark for future research, enabling broader generalizability and long-term impact. Replicating this study in different regions could provide valuable insights for ongoing sustainability efforts. Building upon the foundation set by the Intercollegiate Green Theatre Checklist, a promising future direction would be the development of a specialized checklist, tailored to address the intricacies of NMSC surgery.

Several limitations of this study need to be acknowledged. First, this analysis was confined to only intraoperative carbon emissions from NMSC surgery, and various factors in the broader patient pathway, such as staff and patient transportation, outpatient appointments and post-operative care, were excluded. Moreover, assumptions regarding energy use were necessary due to the absence of individual energy meters for the operating theatre, potentially differing from other institutions. Additionally, as the emission factors in the ICE database are ‘cradle-to-gate’ (that is only include emissions from resource extraction to manufacture of product, before it is transported to the consumer), assumptions were made about the mode and distance of transportation of surgical supplies. The capital costs of machinery, repair, and disposal of reusable surgical instruments were not incorporated in the analysis.

Furthermore, being a single-centre study focusing exclusively on plastic surgery, the generalizability of these findings to other units or specialties involved in skin cancer management may be limited due to variations in operation settings and reconstructive methods used. This study also only included intraoperative carbon emissions; pre-procedure and post-procedure impacts were not included. Lastly, there were no local flap closures during the study interval, so various modelling strategies were used to estimate carbon emissions associated with local flaps. These factors may have resulted in an underestimation of the true impact of NMSC surgery. Interpretation of the results should consider these limitations.

There are various strategies that could be considered to reduce carbon emissions from NMSC surgery. Consumables are the largest contributor to intraoperative carbon emissions. Surgical teams should attempt to minimize the use of disposable consumables and avoid single-use items. Instrument kits should also be streamlined to only include surgical instruments commonly used for NMSC procedures. Surgeons could also encourage NMSC surgery to be performed under local anaesthesia when appropriate, reducing the reliance on general anaesthesia and anaesthetic gases. Lastly, implementing tools such as the Intercollegiate Green Theatre Checklist^75^ can promote greener and more sustainable surgical practices.

This carbon footprinting study offers a crucial insight into the environmental impact of NMSC surgery and emphasizes the need for more sustainable practices within plastic surgery. It is imperative to investigate strategies to reduce carbon emissions linked to building energy use, consumables, and waste generation to mitigate the impact of NMSC surgery. Future data collection is required to improve the power of the study and allow for in-depth analysis of one’s own practices. Surgeons and policymakers should work together to promote sustainability and reduce carbon emissions in healthcare.

## Supplementary Material

zrae084_Supplementary_Data

## Data Availability

The primary data generated and analysed during the course of this research project are available upon reasonable request, with access contingent upon compliance with relevant data protection and confidentiality regulations. Furthermore, the emission factors utilized in this study, including those sourced from the Department for Business, Energy & Industrial Strategy website, the Inventory of Carbon and Energy, WorldStainless Carbon Factors, and Greener NHS emission factors, can be readily accessed through open-access channels. In addition to these resources, the Get Data Out: Skin Tumours data set and the Welsh Cancer Intelligence and Surveillance Unit database are also available through open access.
