# Supplementary material for: Carbon footprint of non-melanoma skin cancer surgery
Source: BJS Open. 2024 Oct 17;8(5):zrae084. doi: 10.1093/bjsopen/zrae084 (PMC11483578; doi:10.1093/bjsopen/zrae084)
Supplement: zrae084_Supplementary_Data [file zrae084_supplementary_data.docx]

**The Carbon Footprint of Non-Melanoma Skin Cancer Surgery**

Ky-Leigh Ang^1, 2^, Matthew Jovic^3^, Ian Malin^2^, Stephen R Ali^1,2,3*^, Iain S Whitaker^1,2,3*^

*Joint Senior Authors

^1^ Oxford University Clinical Academic Graduate School, University of Oxford, Oxford, England

^2^ Welsh Centre for Burns and Plastic Surgery, Swansea, Wales

^3^ Reconstructive Surgery and Regenerative Medicine Research Centre (ReconRegen), Institute of Life Sciences, Swansea University Medical School, Swansea, Wales

**Corresponding author.** Stephen R Ali

**Email:** Stephen.ali@swansea.ac.uk

**Address:** OUCAGS. Room 3A31, The Cairns Library IT Corridor. Level 3, John Radcliffe Hospital (Main Hospital). Oxford. OX3 9DU

Contents

[*Figure S1.* Skin cancer pathway at the Welsh Centre for Burns and Plastic Surgery in Morriston Hospital, Swansea. 3](#_Toc146469300)

[*Figure S2.* Projected mean greenhouse gas emissions trend from NMSC surgery in England and Wales. Upper and lower likely ranges. 4](#_Toc146469301)

[*Figure S3.* Projected mean greenhouse gas emissions trend from NMSC surgery in Wales: A Comparative Analysis of Different Reconstructive Methods. Upper and lower likely ranges. 5](#_Toc146469302)

[*Figure S4.* Projected mean greenhouse gas emissions trend from NMSC surgery in England and Wales: A Comparative Analysis of Different Reconstructive Methods. Upper and lower likely ranges. 6](#_Toc146469303)

[Table S1: Emission factors 7](#_Toc146469304)

[Supplementary table 2: Transport assumptions 9](#_Toc146469305)

[Table S3: Projected greenhouse gas emissions of NMSC in England and Wales 10](#_Toc146469306)

[Appendix S1 12](#_Toc146469307)


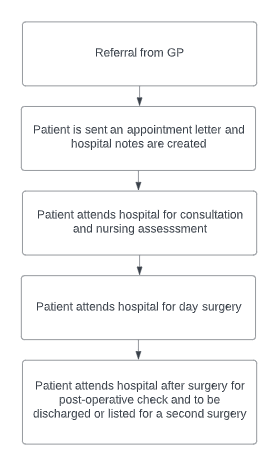


# *Figure S1.* Skin cancer pathway at the Welsh Centre for Burns and Plastic Surgery in Morriston Hospital, Swansea.

*
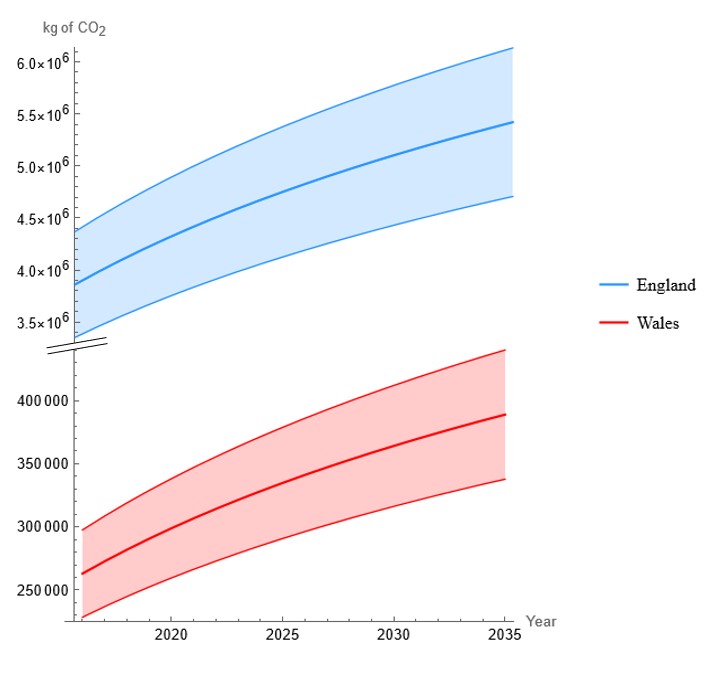
*

# *Figure S2.* Projected mean greenhouse gas emissions trend from NMSC surgery in England and Wales. Upper and lower likely ranges.


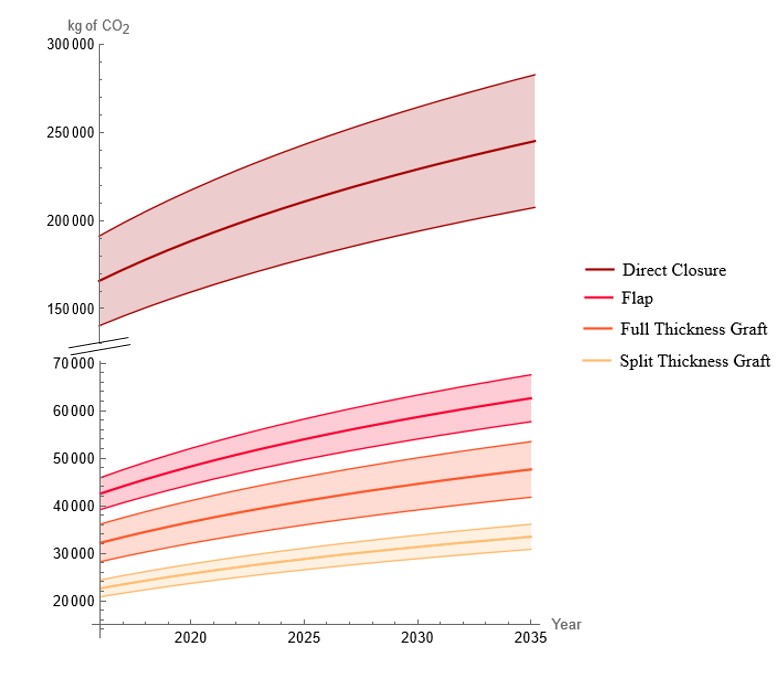


# *Figure S3.* Projected mean greenhouse gas emissions trend from NMSC surgery in Wales: A Comparative Analysis of Different Reconstructive Methods. Upper and lower likely ranges.

*
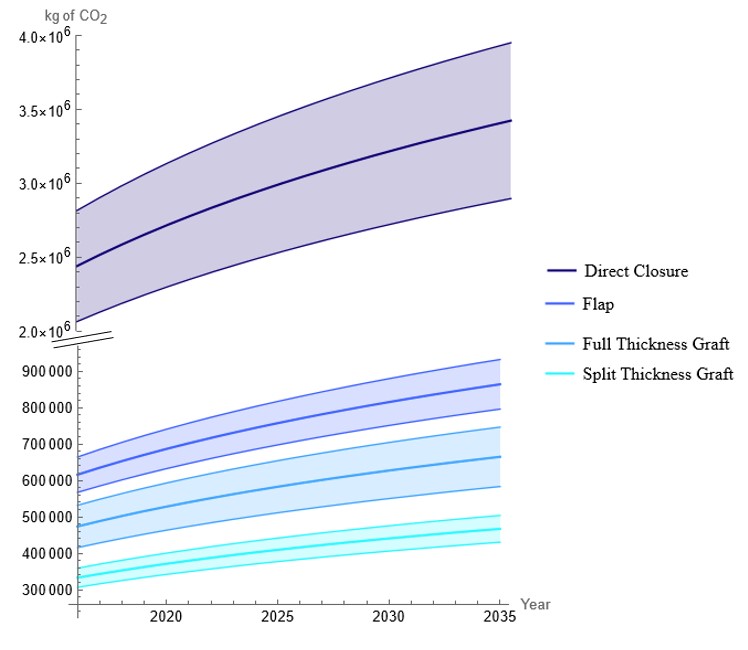
*

# *Figure S4.* Projected mean greenhouse gas emissions trend from NMSC surgery in England: A Comparative Analysis of Different Reconstructive Methods. Upper and lower likely ranges.

# **Table S1: Emission factors**

| Process/ product | | Emission factor | | Emission factor unit | Source |
| --- | --- | --- | --- | --- | --- |
|  |  | Component | Total |  |  |
| Stainless steel | | **6.87** | **6.87** | kg CO_2_e/ kg | WorldStainless[1] |
| Aluminum | | 6.72 | 6.72 | kg CO_2_e/ kg | ICE v3[2] |
| Aluminum foil | | 13.8 | 13.8 | kg CO_2_e/ kg |  |
| Cotton (Fabric) | | 6.78 | 6.78 | kg CO_2_e/ kg |  |
| General polyethylene | | 2.54 | 2.54 | kg CO_2_e/ kg |  |
| General PVC | | 3.10 | 3.10 | kg CO_2_e/ kg |  |
| General Rubber | | 2.85 | 2.85 | kg CO_2_e/ kg |  |
| High density polyethylene (HDPE) resin | | 1.93 | 1.93 | kg CO_2_e/ kg |  |
| Low density polyethylene (LDPE) | | 2.08 | 2.08 | kg CO_2_e/ kg |  |
| Paper | | 1.49 | 1.49 | kg CO_2_e/ kg |  |
| Paperboard | | 1.29 | 1.29 | kg CO_2_e/ kg |  |
| Polypropylene | | 4.49 | 4.49 | kg CO_2_e/ kg |  |
| Polypropylene oriented film | | 3.43 | 3.43 | kg CO_2_e/ kg |  |
| Polyurethane flexible foam | | 4.84 | 4.84 | kg CO_2_e/ kg |  |
| PVC General | | 2.30 | 2.30 | kg CO_2_e/ kg |  |
| Non-sterile glove | | 0.01795 | 0.01795 | kg CO_2_e/ glove | Rizan et al [3] |
| Sterile Gown | | 0.54 | 0.54 | kg CO_2_e/ gown |  |
| Laundry | | 0.46 | 0.46 | kgCO2e/kg | Rizan et al [4] |
| Sterilization | | 0.05 | 0.05 | kgCO2e/instrument in instrument set | Rizan et al [5] |
| Domestic waste | | 0.17 | 0.17 | kg CO_2_e/ kg | Rizan et al [6] |
| Dry mixed recyclable waste | | 0.17 | 0.17 | kg CO_2_e/ kg |  |
| Clinical waste, medicinal contaminated sharps, anatomical waste, medical waste | | 1.07 | 1.07 | kg CO_2_e/ kg |  |
| Infectious Waste | | 0.57 | 0.57 | kg CO_2_e/ kg |  |
| UK electricity | Generation | 0.19 | 0.26 | kg CO_2_e/ kWh | DEFRA/BEIS [7] |
|  | Transmission and distribution | 0.02 |  |  |  |
|  | Generation well to tank | 0.05 |  |  |  |
|  | Transmission and distribution well to tank | 0.004 |  |  |  |
| Water | Supply | 0.15 | 0.42 | kg CO_2_e/ m^3^ |  |
|  | Treatment | 0.27 |  |  |  |
| Transportation | Heavy goods vehicle (diessel, average laden) | 0.11 | 0.14 | kg CO_2_e/ tonne.km |  |
|  | Well to tank | 0.026 |  |  |  |
|  | Cargo ship (container ship, average) | 0.016 | 0.02 | kg CO_2_e/ tonne.km |  |
|  | Well to tank | 0.004 |  |  |  |
|  | Vans (Average) | 0.23 | 0.38 | Kg CO_2_e/ tonne.km |  |
|  | Well to tank | 0.15 |  |  |  |
| Pharmaceuticals | | 0.13 | 0.13 | kg CO_2_e / £ | Centre for Sustainable Healthcare, sourced from Greener NHS Team 2020-21 [8] |
| Surgical sutures | | 0.46 | 0.46 | kg CO_2_e / £ |  |
| Dressings | | 1.54 | 1.54 | kg CO_2_e / £ |  |

# Supplementary table 2: Transport assumptions

| Country of Origin | Port | Assumed distance for shipping to London Gateway Port (km) [9] |
| --- | --- | --- |
| China | Shanghai | 19,303 |
| Egypt | Damietta | 5,952 |
| France | Le Harve | 347 |
| Germany | Hamburg | 739 |
| Malaysia | Port Klang | 15,026 |
| Mexico | Lázaro Cárdenas | 11,868 |
| Thailand | Bangkok | 16,950 |
| Netherlands | Rotterdam | 259 |
| Spain | Valencia | 3229 |
| United States of America | New York | 1990 |
| India | Mundra | 11513 |

Assumed ports and distances to the UK. We included 200km of travel by road via heavy goods vehicles both within the country of origin and in the UK and an additional 20km via courier.

# Table S3: Projected greenhouse gas emissions of NMSC in England and Wales

|  | **kg CO2 (95% CI)** | | | | |
| --- | --- | --- | --- | --- | --- |
| **Year** | Full Thickness Graft | Split Thickness Graft | Flap | Direct Closure | Total |
| 2016 | 473707 (415554 - 531860) | 332832 (306583-359080) | 615888 (567316-664459) | 2438768 (2063573-2813963) | 3861196 (3353027-4369364) |
| 2017 | 488520 (428548 - 548491) | 343239 (316170-370308) | 635123 (585034-685211) | 2515025 (2128098-2901951) | 3981907 (3457851-4505963) |
| 2018 | 473707 (415554-531860) | 353051 (325208-380894) | 653258 (601739-704777) | 2586921 (2188933-2984909) | 4095716 (3556681-4634752) |
| 2019 | 488519 (428548-548490) | 362333 (333757-390908) | 670413 (617541-723284) | 2654930 (2246479-3063381) | 4203371 (3650166-4756576) |
| 2020 | 502484 (440799-564170) | 371138 (341868-400408) | 686687 (632532-740842) | 2719450 (2301073-3137826) | 4305502 (3738855-4872150) |
| 2021 | 515694 (452387-579002) | 379514 (349583-409444) | 702167 (646791-757543) | 2780820 (2353002-3208639) | 4402650 (3823216-4982084) |
| 2022 | 528227 (463381-593073) | 387500 (356940-418059) | 716927 (660387-773467) | 2839336 (2402515-3276157) | 4495277 (3903652-5086903) |
| 2023 | 540147 (473838-606457) | 395130 (363969-426292) | 731031 (673378-788683) | 2895250 (2449826-3340673) | 4583786 (3980511-5187061) |
| 2024 | 551513 (483809-619218) | 402436 (370699-434174) | 744534 (685817-803251) | 2948783 (2495124-3402442) | 4668528 (4054099-5282957) |
| 2025 | 562374 (493336-631412) | 409444 (377154-441735) | 757486 (697748-817225) | 3000131 (2538573-3461690) | 4749810 (4124683-5374937) |
| 2026 | 572773 (502458-643087) | 416177 (383356-448999) | 769930 (709210-830650) | 3049465 (2580317-3518614) | 4827903 (4192498-5463309) |
| 2027 | 582746 (511208-654285) | 422656 (389323-455988) | 781905 (720240-843569) | 3096937 (2620485-3573389) | 4903049 (4257753-5548345) |
| 2028 | 592329 (519614-665044) | 428899 (395074-462724) | 793444 (730869-856018) | 3142682 (2659193-3626172) | 4975462 (4320635-5630289) |
| 2029 | 601550 (527703-675397) | 434923 (400623-469223) | 804578 (741125-868030) | 3186822 (2696542-3677102) | 5045333 (4381310-5709357) |
| 2030 | 610436 (535498-685374) | 440743 (405984-475502) | 815334 (751033-879635) | 3229465 (2732624-3726306) | 5112836 (4439928-5785744) |
| 2031 | 619009 (543019-695000) | 446372 (411169-481574) | 825738 (760616-890859) | 3270710 (2767524-3773896) | 5178124 (4496623-5859626) |
| 2032 | 627292 (550285-704300) | 451822 (416189-487454) | 835811 (769895-901727) | 3310645 (2801315-3819975) | 5241340 (4551519-5931162) |
| 2033 | 635304 (557313-713295) | 457104 (421055-493153) | 845574 (778889-912260) | 3349351 (2834067-3864636) | 5302611 (4604725-6000497) |
| 2034 | 643061 (564118-722004) | 462229 (425776-498682) | 855046 (787614-922479) | 3386902 (2865840-3907964) | 5362052 (4656342-6067761) |
| 2035 | 650579 (570713-730445) | 467205 (430359-504051) | 864244 (796086-932402) | 3423364 (2896693-3950036) | 5419770 (4706464-6133076) |

**Reference**

1. WorldStainless. *Stainless Steels and CO2: Industry Emissions and Related Data*. 2022 04 April 2023]; Available from: <https://www.worldstainless.org/about-stainless/environment/stainless-steels-and-co2-industry-emissions-and-related-data/#:~:text=industry's%20CO2%20emissions.-,Scope%201%20Emissions,tonne%20of%20stainless%20steel%20produced>.

2. Jones, C. and G. Hammond, *Inventory of Carbon and Energy v3.0*. 2019.

3. Rizan, C., M. Reed, and M.F. Bhutta, *Environmental impact of personal protective equipment distributed for use by health and social care services in England in the first six months of the COVID-19 pandemic.* Journal of the Royal Society of Medicine, 2021. **114**(5): p. 250-263.

4. Rizan, C., *Mitigating the carbon footprint of products used in surgical operations*, in *Brighton and Sussex Medical School*. 2023.

5. Rizan, C., et al., *Minimising carbon and financial costs of steam sterilisation and packaging of reusable surgical instruments.* British Journal of Surgery, 2022. **109**(2): p. 200-210.

6. Rizan, C., et al., *The carbon footprint of waste streams in a UK hospital.* Journal of Cleaner Production, 2021. **286**: p. 125446.

7. Department for Business, Energy & Industrial Strategy and Department for Energy Security and Net Zero *UK Government GHG Conversion Factors for Company Reporting* 2022.

8. Centre for Sustainable Healthcare. *Carbon Footprinting: Carbon emissions factors specific for healthcare settings*. [cited 2023 04 April]; Available from: <https://www.sustainablehealthcarelearning.com/>.

9. Pier2Pier. *Pier2Pier*. [04 April 2023]; Available from: <https://www.pier2pier.com/Co2/>.

# Appendix S1

***Methods for analysis focused on water used in a traditional surgical hand scrub.***

The World Health Organisation defines a standard preoperative aqueous scrub as 2 minutes of scrubbing with soap washing each side of arm from wrist to elbow for 1 minute, amounting to 2 minutes of continuous water use per scrub [1]. We aim to assess the amount of water used during a surgical hand scrub and to determine the percentage of water actively used during the procedure.

Surgical scrubbing was observed in one operating theatre at the Welsh Centre for Burns and Plastic Surgery, Morriston Hospital, Swansea and included doctors and nurses who routinely performed surgical scrubs. Participants were not informed of the study's purpose to avoid altering their behaviour. Thirty-five separate surgical scrubs were observed and and the length of time for which the tap was turned on was recorded with a stopwatch.

To determine the total amount of water used during a scrubbing episode, the volume of water dispensed from taps set to a medium flow rate was measured for 1 min. The volume of water was calculated using a standard density of 997 kg/m³. This process was repeated thrice, and the mean flow was determined. The total time of water flowing, and time of water being used for each surgical scrubbing episode were recorded. The time of water flowing was measured from when the tap starts running until closure of the tap. The time of water flowing was measured from when the tap starts running until it is turned off, and the time of water being actively used was when water was being actively used. We also calculated the carbon footprint of three surgical scrubbing episodes using the Department for Business, Energy & Industrial Strategy (BEIS) Greenhouse Gas Conversion Factors for Company Reporting [2].

The surgical team often consisted of one registrar with either a consultant or a junior trainee. The rest of the team consisted of one scrub nurse and one theatre nurse. Carbon emissions from water use are reported based on 3 surgical scrubs.

***Results***

The average time for which the tap was turned on during surgical scrubbing was 1 min 52 s (maximum: 3 min 7 s; minimum: 1 min 2 s; SD: 29 s). The flow rate of the tap was approximately 7.13 L/min, resulting in an average of 13.30 L of water used per scrub.

The mean time of water being actively used during the procedure was 27 s (maximum: 1 min 2 s; minimum: 10 s; SD: 3.76 s). The mean percentage of water actively used per surgical scrubbing was 23.61% (maximum: 37.69%; minimum: 51.33%; SD: 2.25%).

Based on these findings, it was estimated that 0.006 kgCO2eq per surgical scrub and three surgical scrubbing episodes would produce approximately 0.02 kgCO2eq. These findings suggest that there may be opportunities to reduce water usage during surgical hand scrubbing without compromising hygiene practices.


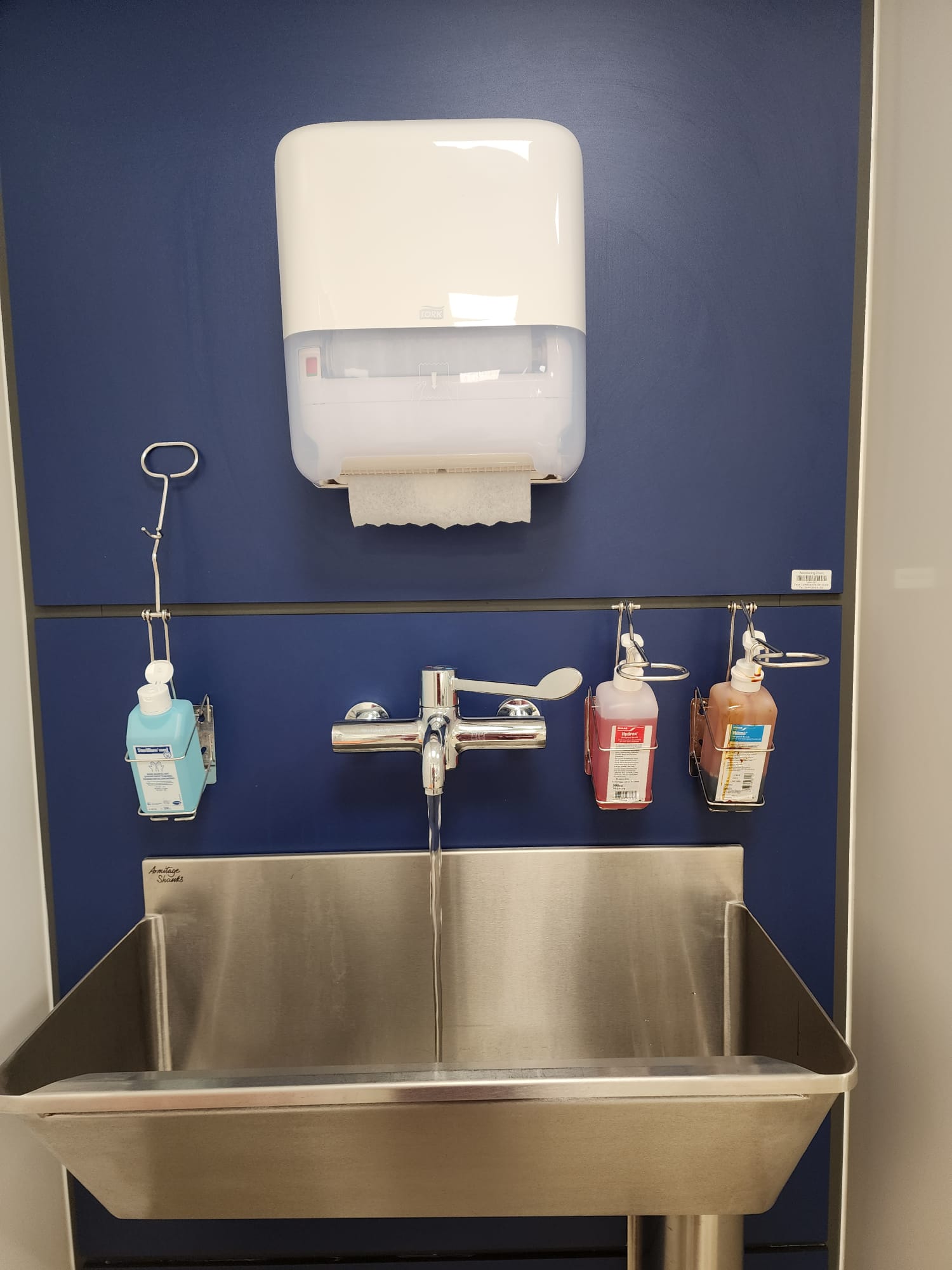


*Supplementary results figure 1:* The ‘elbow on’ variety of tap used at our treatment center at The Welsh Centre for Burns and Plastic Surgery, Morriston Hospital.


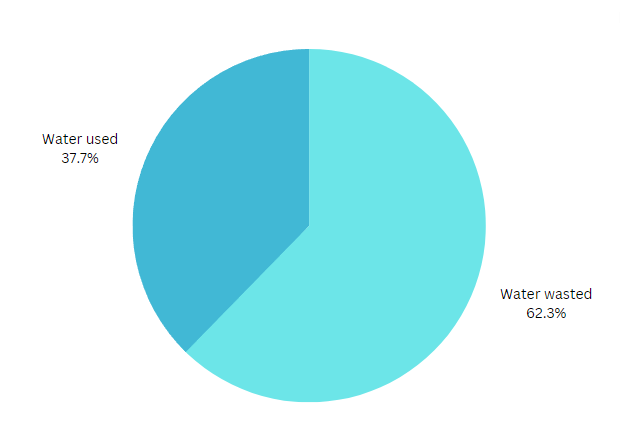


*Supplementary results figure 2:* Percentage of water used and water being wasted per episode of surgical scrub.

**Reference**

1. World Health Organization. *WHO Guidelines on Hand Hygiene in Health Care: Clean Care is Safer Care*. 2009; Available from: <https://apps.who.int/iris/bitstream/handle/10665/44102/9789241597906_eng.pdf>.

2. Department for Business, E.I.S., *Greenhouse gas reporting: conversion factors 2022*. 2022.
